# Global Brain Drain: How Can the Maslow Theory of Motivation Improve Our Understanding of Physician Migration?

**DOI:** 10.3390/ijerph16071182

**Published:** 2019-04-02

**Authors:** Lena Dohlman, Matthew DiMeglio, Jihane Hajj, Krzysztof Laudanski

**Affiliations:** 1 Department of Anesthesia, Critical Care, and Pain Medicine, Massachusetts General Hospital, Boston, MA 02114, USA; Ldohlman@hotmail.com; 2 DO/MBA Student, Philadelphia College of Osteopathic Medicine, Philadelphia, PA 19131, USA; matthewdi@pcom.edu; 3 Department of Cardiology, Penn Presbyterian Medical Center, Philadelphia, PA 19104, USA; Jihane.Hajj@uphs.upenn.edu; 4 Department of Anesthesiology and Critical Care, Hospital of the University of Pennsylvania, Philadelphia, PA 19104, USA; 5 Leonard Davis Institute of Healthcare, University of Pennsylvania, Philadelphia, PA 19104, USA; 6 Global Health Initiative, University of Pennsylvania, Philadelphia, PA 19104, USA

**Keywords:** brain drain, physician migration, low- and middle-income countries, physician workforce, Maslow theorem

## Abstract

The migration of physicians from low-resource to high-resource settings is a prevalent global phenomenon that is insufficiently understood. Most low-income countries are severely understaffed with physicians, and the emigration of the already limited number of physicians to other countries can significantly reduce access to healthcare in the source country. Despite a growing interest in global capacity building in these countries by academic and non-governmental organizations in high-income countries, efforts to stem physician migration have been mostly unsuccessful. The authors reviewed the current literature for the motivational factors leading to physician migration in the context of Maslow’s hierarchy of human needs. Our study found that financial safety needs were major drivers of physician emigration. However, factors related to self-actualization such as the desire for professional development through training opportunities and research, were also major contributors. These findings highlight the multifactorial nature of physician motivations to emigrate from low-resource countries. Maslow’s Theory of Motivation may provide a useful framework for future studies evaluating the concerns of physicians in low-income countries and as a guide to incentivize retention.

## 1. Introduction

The benefits of physician migration to academic centers in high resource countries are something the authors have personally experienced and appreciated. However, in our volunteer efforts to train and build the physician workforce in low-resource countries, we have also seen the resulting damage to healthcare delivery when medical personnel migrate out of their country. We sought to improve the understanding of physician migration from lower-resource countries by finding a common language on motivating factors for more consistent data collection. We mainly wanted to investigate whether financial need is the primary driver of physician migration or if other motivating factors also play important roles. It is essential to carefully examine the literature using a widely accepted framework because a comprehensive assessment of current knowledge on physician motivations is necessary for developing initiatives to counteract migration. However, existing literature on motivations of physician emigration is descriptive and is missing a theoretical framework that could be used to build a database that is consistent across different political and social environments.

**Figure 1:**
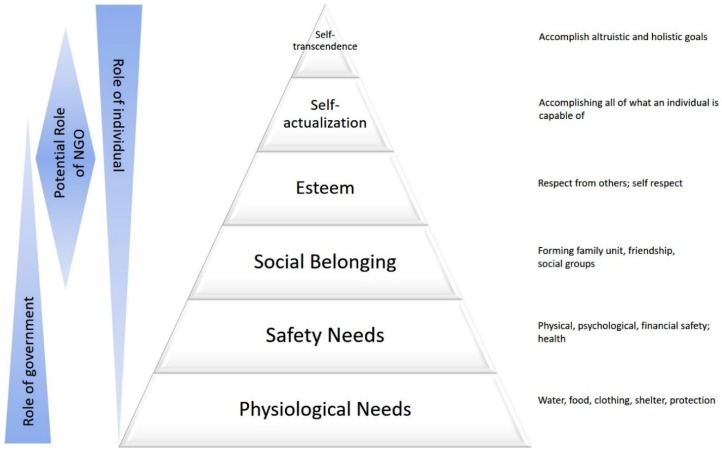
Maslow’s theorem of self-actualization and motivation stresses the hierarchical nature of needs governing the behavior of individuals [32,33].

We hypothesized that Maslow’s hierarchy of human needs would be a useful framework for collecting data on physician motivations to migrate. Abraham Maslow was an American psychologist who proposed a hierarchy of human needs that he believed determined how people are motivated. He believed that human needs fall into five categories, that the needs in the lower categories are stronger drivers of motivation than higher ones, and that they must be at least partly satisfied before a person will be motivated to work towards the higher categories. The five categories were the following: physiological needs, safety needs, social needs, esteem needs, and self-actualization [1,2] (Figure 1). Maslow’s theory of motivation has remained influential, particularly in psychology and business management, as a tool to understand people’s motivation for behavioral change. This theorem is also widely known by medical and non-medical professionals and can be used as a common language when discussing the issue of migration. The Maslow model may provide a framework to understand what factors are most important to physicians and how they come to their decision to emigrate to higher-resource countries.

The worldwide shortage and maldistribution of healthcare workers are at a critical stage and is threatening the sustainability of health systems [3]. The World Health Organization (WHO) estimated in 2013 that there was a global shortage of 7.2 million healthcare providers [4]. This shortage is predicted by some to reach 15 million by 2030 [4,5]. The need for more workers trained in evidence-based medicine is especially severe in low- to medium-income countries (LMICs) where 83 countries are unable to meet the basic minimum threshold of 23 trained health professionals per 10,000 people [4]. This undersupply is made worse by migration of large numbers of educated health workers from LMICs to high-income countries (HICs), where there are also shortages, but the impact on LMICs is disproportionally more severe [3,6,7].

“Brain drain” is particularly a problem in Africa, which has 25% of the global disease burden but only 3% of the global health workforce [8]. Some African countries lose up to 70% of their health workforce to migration, and an estimated one-fifth of African-born physicians work in HICs [9,10]. The loss of trained health personnel from areas where health systems are already stressed to their limits leave the remaining professionals overwhelmed and demoralized and can result in a critical lack of services [7,11,12,13,14,15]. An analysis published in 2017 found significant decreases in maternal, neonatal, and under-5 mortalities for every increase of one physician per 1000 population in 208 countries, supporting the importance of an adequate physician workforce [16]. The loss of physicians to migration is particularly expensive for governments and medical communities in LMICs because of the number of years and the cost it takes to train them, and this is the reason we chose to particularly focus on physician migration [17,18]. One analysis of net loss to nine LMICs in Africa, from the outflow of their trained physicians currently working in HICs, was estimated at $2.17 billion [19]. High resource countries in North America, Western Europe, and Australia have actively recruited trained healthcare workers from low-resource countries and benefit by not having to invest in subsidizing their full education [7]. Nearly one-quarter of active physicians in the United States (U.S.) are international medical graduates (IMGs), and more than 50% of IMGs are from LMICs [10]. This migration pattern puts a significant financial as well as health service stress on impoverished countries, while high-resource countries benefit [12,20,21,22,23,24,25]. Internal migration from high-need rural and poor areas to urban areas is also a problem in nearly all countries and may contribute to the unnecessary rise of health care costs in areas where health professionals are excessively concentrated [26]. Several studies in HICs have found that healthcare workers that grew up and/or trained in rural areas are easier to recruit to remote areas. Rural area retention of healthcare workers in HICs was also found to be influenced by working conditions, environment, and opportunities for professional advancement. This emphasizes the fact that financial incentives are not the only factors playing a role in provider distribution [27]. Whether these same factors play a role in LMICs is unclear since few studies have been done in these countries on what motivates internal migration of physicians. Obtaining data on the role of financial versus other motivations as reasons for emigration from LMICs is critical for establishing future initiatives to manage the brain drain problem.

Despite limited information on the factors influencing physician migration, there have been some efforts to counteract the outflow of doctors from LMICs to HICs [17,20,28,29]. Some organizations and policymakers have proposed repayment programs by HICs for the loss of trained workers from low-resource countries [30]. In 2010, the World Health Organization (WHO) published the Global Code of Practice on the International Recruitment of Health Personnel [31]. The international community hoped to pressure HICs into voluntarily decreasing the recruitment attempts of health personnel away from the LMICs countries in which they were trained. There is no evidence that the overall rate of physician emigration from LMICs has decreased after these efforts, suggesting that current approaches need reassessment [11,32,33]. An improved understanding of unmet needs of physicians who migrate may lead to more effective approaches.

## 2. Methods

**Figure 2:**
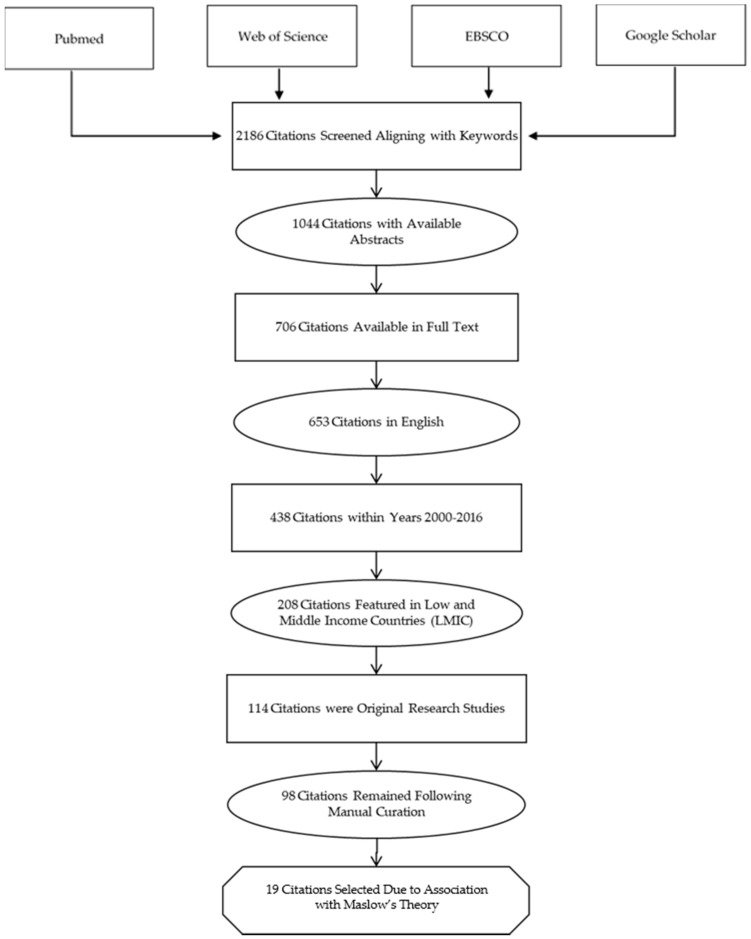
The selection, inclusion, and exclusion criteria are illustrated through a flow chart. Inclusion criteria involved abstracts and full texts that were readily available. From this pool of citations, the inclusion criteria mandated the articles be within the date range of 2000–2016. Articles included were representative of low- and middle-income countries (LMICs). Redundant articles and case reports were excluded to yield a total of 19 articles.

We surveyed PubMed, Web of Science, EBSCO MEDLINE, and the Google Scholar scientific database. We used the terms “physician emigration,” “physician migration,” and “physician brain drain” as keywords. We curated the database manually to remove citations about the migration of physicians within HICs. We focused our review on physicians due to the considerable loss of return on investment for LMICs described in previous literature [19]. We limited the search to original research, peer-reviewed studies published between 2000 and 2016. We excluded non-English language articles and those we could not access. Selection process resulted in 105 publications focusing predominantly on countries in Africa and Asia (Figure 2). There were only a few articles that focused on LMICs in the Middle East and Europe. Nineteen articles contained collected responses from questionnaires searching for motivations for emigration. (Table 1). The articles represent a convenience sample. Several authors published a series of updated manuscripts [34,35,36,37]. Others followed their original work up with a more editorialized version of their prior work or reviews [38,39]. We included only representative papers to improve the clarity of the table. The remaining articles reviewed were general discussion papers on physician migration.

## 3. Results

Nineteen articles satisfied the search criteria and gave specific answers to questionnaires asking for motives to emigrate from the country of training to higher-income countries. In these articles, basic food and shelter needs were mentioned twice, physical and financial security needs 12 times, a need for social belonging twice, a desire to improve educational or professional opportunities (self-esteem) 13 times, and self-actualization or “being all you can be” 15 times (Table 1) [27,31,36,40,41,42,43,44,45,46,47,48,49,50,51,52,53,54,55,56,57,58].

The first level of Maslow’s hierarchy, the need for food and shelter, is mentioned only twice as a motivator of emigration in any of the studies reviewed [43,56]. This finding may be due to publication bias. The need for food and shelter by physicians would be expected only to occur in a war zone, or in a disaster area. It would be unlikely that research on physician migration would be carried out in these areas since there would be no medical providers in the first place. Additionally, any research in war zone is inherently difficult. However, the second level, which includes the need for personal safety and security, is frequently mentioned as a reason for emigration, especially in politically unstable or socially intolerant countries. The need for freedom of expression, not being prosecuted because of sexual preferences, religion, or political beliefs, and the ability to provide financially for your family are examples of these security needs. Domestic terrorism and religious intolerance are mentioned as motivators for emigrating from Pakistan [40,45,58]. Women seem especially sensitive to this issue as they are frequently targeted [40]. Many specialist physicians practicing in war-torn Iraq between 2004 and 2007 left practice in urban tertiary care hospitals where the risks of personal injury were highest [59,60]. In Lebanon, political instability resulted in nearly 100% of physicians wishing to leave the country [36,37]. A recent study of South African health care providers found that racial tensions and concerns over increased criminal activity were stronger determinants of seeking work internationally than other factors, including financial remuneration [61]. The threat of worsening disease transmission, such as the AIDS epidemic manifesting in sub-Saharan Africa, has also correlated with increased migration [62]. Even in regions where infrastructure support is provided (e.g., the People’s Republic of China), doctors’ personal safety is threatened by violence from angry patients and is driving physicians away [63]. Safety seems to be a fundamental need and should be considered a basic foundation for preventing migration.

The second level of Maslow’s hierarchy also includes the need for financial security. In half the studies in which medical personnel’s opinion on emigrating was elicited, a desire for improved financial earnings was explicitly mentioned as a driver for emigration (Table 1) [31,40,41,45,48,52,53,56,59]. Other papers also provided evidence that improving financial conditions locally can decrease emigration. A study by Okeke analyzed the consequences of a government-offered bonus stipends paid to physicians in Ghana [38]. The Additional Duty Hours Allowance compensated physicians working over 40 hours per week, providing a potential 150% increase to base salaries, while also improving access to care from physicians. The six-year incentive program resulted in an approximately 10% drop in Ghanaian physicians working outside the country [38]. In Ethiopia, a multipronged approach was implemented to improve physicians’ financial security through a land donation, transportation support, tax abatement, and salary support [11,64]. Since this program began, the retention of doctors has increased, suggesting the moderate success of such strategies. However, above a certain income threshold, financial incentives are not the most important factors in the decision to emigrate in at least one study [9]. Okeke has provided evidence that financial motivations for leaving one’s home country lessen with increased Gross Domestic Product (GDP) and that other factors can be more powerful motivators [38,39]. Concomitantly, it has been shown that emigration rates are higher in sub-Saharan African countries with modest, but not the lowest, GDP (Nigeria, Ghana, and South Africa) [44,65]. There are several editorials in the literature that discuss the potential, non-financial underlying reasons of migration but we did not include them in our analysis. In the following paragraphs we will instead take a fresh look at these factors based on our analysis of the data in the papers we reviewed.

Maslow’s third level, the need for social acceptance, was found primarily to serve as a deterrent for migration. Having strong personal and family roots in a native country are good predictors of retention, and this was one of the primary reasons voiced by students from Malawi and Nigeria for wanting to practice locally [44,66]. Family ties were more often cited by female physicians as a reason for not leaving their country of origin [41,45,64]. Male and young physicians expressed the desire to emigrate more often than females and older physicians [36,41,45,64]. Having relatives or friends abroad or being a dual citizen made migration more likely [31,61,67]. However, increasing globalization of healthcare workers and improved communication associated with this globalization appears to facilitate migration [41,68].

Maslow’s fourth level, the need for self-esteem or desire for educational and professional development opportunities, was mentioned 13 times in the questionnaires studied (Table 1) [31,41,42,45,49,50,51,52,53,54,56]. The desire for professional development after medical school was found especially among medium-income (Croatia) and low-income countries (e.g., Tanzania, Ghana, Malawi) with satisfactory geopolitical stability and safety [42,49,52]. The same drive is also seen in medical students from several sub-Saharan African countries, as well as Nepal and the Philippines [34,35,66,67,69,70]. Most of these future physicians’ plan for a temporary departure, and their goal is to acquire new educational knowledge and skills that cannot be obtained at home. A minority of physicians had intentions for a permanent relocation. Among this minority, the motivation for access to quality training opportunities was there, but the desire for a higher standard of living in HICs also differentiated these physicians [27,37,53]. Bidwell et al. show that experience working in a different healthcare system is a major factor in driving a physician’s emigration from South Africa even if they have access to high levels of training in their home countries [61]. A similar observation has been made in Ethiopia [11,44]. This is an important consideration for local governments. Providing training opportunities may reduce brain drain, but it will not stop it completely.

Maslow’s fifth level, self-actualization or “being all you can be,” was the most commonly mentioned reason for emigrating in the questionnaires included in this review. Physicians mentioned the need to improve “research opportunities” and “career opportunities” and to “excel professionally” (Table 1) [31,40,41,42,44,45,47,48,49,51,52,53,54,56,57]. One survey involving Croatian final-year medical students found that respondents were more inclined to emigrate for opportunities to excel professionally and acquire new experiences than for financial prosperity [42]. Job satisfaction is directly linked to the decision to emigrate for Ghanaian physicians [70].

## 4. Discussion

Physician migration from LMICs to higher-resource countries is a poorly understood phenomenon that has significant consequences on health care in all countries. Our review of the literature has found that motivating factors vary somewhat from country to country, but many are shared and can be categorized into Maslow’s hierarchy of human needs [32,33]. The factors that primarily seem to influence migration include a need for personal security (Level 2), social acceptance (Level 3), a need for improved educational and professional opportunities or self-esteem (Level 4), and a need for self-actualization (Level 5). Maslow’s first level, the basic physiologic need for food and shelter is mentioned only twice [43,59,60]. This may imply that, even in LMICs, a physician’s salary is adequate to provide for basic food and shelter. Unless physicians are in an active war or refugee situation, they are unlikely to be searching for basic food and shelter, so these are less likely to be motivating factors for migration [63]. Improving the physical safety of physicians is a complex problem that cannot be dealt with easily because it is intertwined with the socio-political situation of countries. International non-governmental organizations (NGOs) may be able to help improve physical security through social and political pressure, but most are not structured to provide adequate local support.

The effect of financial security on emigration has been more often studied, perhaps because an increase in income potential has traditionally been assumed to be the primary driver of brain drain [38,41,48]. Our results confirmed that the need for financial security is a common factor motivating physicians to want to emigrate (Table 1). Some articles reviewed supported the idea that increased health worker stipends and other economic incentives can slightly reduce emigration rates [38,39]. However, the brain drain is undoubtedly more than just a salary issue. Extreme financial disincentives may force a medical professional out of medicine altogether [71]. One study demonstrated that a 1% decline in gross domestic product decreases the medical workforce by approximately 3% [17]. Studies by Asongu suggest that brain drain is variably influenced by the gross national product [9]. However, above a certain financial threshold, the financial factor is removed as the most important in the decision to emigrate, an observation confirmed by another study [38,39]. In more prosperous countries (South Africa, Lithuania, Croatia, and Nigeria), financial incentives have lower impacts on emigration rates, and other needs, such as professional development opportunities, prevail [42,66,67]. Studies in Ethiopia showed similarly that a financial incentive is not the predominant factor [11,44,64,71]. We speculate that, in LMICs countries with a relatively increased GDP, other needs are centered at a higher level of Maslow’s theory such as self-actualization.

The need for social belonging was infrequently mentioned as a motivator for emigration and only in situations where relatives or other social supports were present in the destination country [11,44]. Physicians are motivated to remain with social supports, all else being equal, and social supports at home can, therefore, be an inhibitor for emigration [8,47,48]. After providing for basic safety and financial security, and if social supports exist at home, an LMIC can best motivate physicians to remain in their country of training by making their practice more financially secure and professionally satisfying.

Our review found that non-monetary factors are important drivers of migration. Once their basic safety and financial security needs are met, physicians seek to satisfy their need for self-esteem and self-actualization through improved professional development, advancements, and opportunity to pursue research [47,49,59,66]. Especially in middle-income countries with stable geopolitical situations, the lack of training and professional opportunities are the main drivers of emigration [36,37]. When physicians from LMICs are provided with temporary training opportunities abroad and return afterward, they can bring new expertise and ideas that may improve standards [40,47,49]. However, the temptation to stay abroad due to improved income, resources, and opportunities may be high. Providing medical education in the home country has an advantage over providing training overseas by tailoring experiences to local conditions, strengthening participants’ ties to the native country, and not providing an easy opportunity to emigrate [37,47,72,73,74]. Increasing desirable employment opportunities via local and international partnerships is also critical for retention, and several large programs are aimed at job creation [75,76]. Financial incentives contribute to emigration decisions, but they cannot be considered the primary drivers. In countries where the financial needs of physicians are met, the effective prevention of brain drain should address this next level of need—better education and professional opportunities. Collaboration between national medical societies, university training programs, and NGOs have expanded the efforts to globalize medical care and bring specialty training to developing areas [76,77]. These efforts bring the “outside” world to local communities, allowing for a sense of participation and self-actualization being nurtured locally. Improving physicians’ professional satisfaction in LMICs should improve retention. With greater numbers of practitioners, there should be more time for self-sustaining education and professional development. These are some tentative conclusions that might guide future attempts to slow the brain drain from high-need, low-resource countries. However, more consistent data would improve our understanding of physician emigration. Maslow’s hierarchy of human needs may serve as a source of standard language for data collection on motivations for physician emigration. Better data should aid in developing and studying methods to increase the retention of medical professionals in their country of training.

## 5. Conclusions

Maslow’s theory of motivation provided a useful framework to highlight the multifactorial nature of physician migration from LMICs. Current studies indicate that the development of comprehensive incentive programs and better academic infrastructure could be useful avenues to promote physician retention. This review also highlights the need for future studies evaluating physician migration and mechanisms of retention.

## Figures and Tables

**Table 1:** Application of Maslow’s Theory to selected studies on physician migration.

Ref.	Study Population	Study Sample	Percentage with the Intention to Leave	Reasons to Emigrate	The Equivalent on Maslow Theory
[51]	Ireland	1519 (medical trainees)	88%	career opportunities (85%), working conditions (83%), lifestyle (80%).	Self-actualization Esteem
[47]	Egypt	940 (students)	85.7% (81.8% plan to return)	better research opportunities (mean 4.74) working conditions (mean 4.64)	Self-actualization
[48]	Uganda	251	44.6%	“*doctors are paid a high salary abroad,* *safe working environment, and* *the desire to continue academics.*”	Safety needs Self-actualization Esteem
[52]	Bangladesh, Ethiopia, India, Kenya, Malawi, Nepal, Tanzania and Zambia	3156 (medical and nursing students)	28%	educational opportunities, monetary opportunities	Safety needs Self-actualization Esteem
[49]	Malawi	12 (medical students)	All participants intended to work in Malawi in the long term, after a period outside the country.	“[T] his *was in the pursuit of postgraduate study rather than higher salaries. In general, medical students and young doctors were enthusiastic about working at district level, although this is curtailed by their desire for specialist training and frustration with resource shortages*.”	Self-actualization Esteem
[42]	Croatia	232	53% for specialty (26%) or subspecialty (27%) training	excel professionally (38%), prosper financially (17%), and acquire new experiences and international exposure (26%).	Safety needs Self-actualization Esteem
[44]	Ethiopia	600 (medical students)	53% of the students	*“improving the quality of education and career choice satisfaction, creating conducive training and working conditions.”*	Safety needs Self-actualization Esteem
[53]	Pakistan	275 (medical students)	60.4%; 14.2% intended to return to Pakistan immediately after training; 10% never intended to go back to Pakistan or wished to stay abroad temporarily (37%).	the impact of training on future career (mean score 8.20 ± 2.3), financial conditions of doctors (mean score 7.97 ± 2.37), and job opportunities (mean score7.90 ± 2.34).	Safety needs Self-actualization Esteem
[36]	Lebanon	425 (medical students)	95.5%; 25.1% respondents intended to return to Lebanon directly after finishing training abroad; 63.8% intended to return to Lebanon after working abroad temporarily for a varying number or years; 10.6% intended to never return to Lebanon.	training perspective	Self-actualization
[56]	Democratic Republic of Congo, Kenya, Nigeria, Tanzania and Uganda	990 (medical students)	Many students (40%) planned to train abroad.	Career-related factors favoring retention in Africa were career options and the quality and availability of training opportunities. The top personal factors for staying in Africa were a desire to improve medicine in Africa, personal safety, social conditions, and family issues. The top career-related factors favoring relocation outside Africa were remuneration, access to equipment and advanced technology, career and training opportunities, a regulated work environment, and the politics of health care in Africa. The top personal factors favoring relocation outside Africa were personal safety, the opportunity for experience in a different environment, social conditions, and greater personal freedom.	Self-transcendence Self-actualization Social belonging Safety needs Food shelter
[55]	Ghana	282	64.9% had considered emigrating after graduation.	Consideration of emigration was predicted by having lived abroad but never in a rural area (OR: 3.39, 95%CI: 1.15–9.97).	Social belonging
[54]	Nepal	265 (student, interns, and house officers)	40% of students, 58% of interns, and 48% of house officers	Improving career opportunities or the working environment of the doctor could make the profession more attractive.	Self-actualization Esteem
[46]	India	260 (medical students)	59% intend to leave for training.	While more than 60% perceived greater professional opportunities in the United States than in India, approximately 75% were concerned that the United States had become less welcoming after the terrorist attacks of 9/11, and similar numbers were concerned about the examination administered by the Educational Commission on Foreign Medical Graduates. Conversely, the majority of respondents felt that opportunities for physicians in India were improving.	Self-actualization, Safety needs
[41]	Sri Lanka	374 (students, pre-interns)	23.8%	better quality of life, better earnings, and more training opportunities	Self-actualization Esteem Safety needs
[43]	Iraq	1243 (physicians)	61% left the country.	safety, security, poor financial conditions	Safety needs Food and shelter
[45]	Pakistan	240 (students, interns)	54%	48%: postgraduate education, 35.2%: economic prospects, 92.5%: weak medical system, 78.5%: religious intolerance	Safety needs Self-actualization Esteem
[40]	Pakistan	323 (students)	60.4%	lucrative salary, quality of training, job satisfaction better way of life, relatives, and domestic terrorism	Self-actualization Esteem Social belonging Safety needs
[31]	Romania	957 (students)	84.7%	earning potential, better life	Safety needs Self-actualization Esteem
[57]	Mongolia	39 (physicians)	26%	insufficient equipment supply training funding	Self-actualization
